# MR‐guided proton therapy: Impact of magnetic fields on the detector response

**DOI:** 10.1002/mp.14660

**Published:** 2021-04-03

**Authors:** Hermann Fuchs, Fatima Padilla‐Cabal, Lukas Zimmermann, Hugo Palmans, Dietmar Georg

**Affiliations:** ^1^ Division of Medical Physics Department of Radiation Oncology Medical University of Vienna 1090 Vienna Austria; ^2^ Division of Medical Physics MedAustron Ion Therapy Center 2700 Wiener Neustadt Austria; ^3^ National Physical Laboratory TW11 0LW Teddington United Kingdom

## Abstract

**Purpose:**

To investigate the response of detectors for proton dosimetry in the presence of magnetic fields.

**Material and Methods:**

Four ionization chambers (ICs), two thimble‐type and two plane‐parallel‐type, and a diamond detector were investigated. All detectors were irradiated with homogeneous single‐energy‐layer fields, using 252.7 MeV proton beams. A Farmer IC was additionally irradiated in the same geometrical configuration, but with a lower nominal energy of 97.4 MeV. The beams were subjected to magnetic field strengths of 0, 0.25, 0.5, 0.75, and 1 T produced by a research dipole magnet placed at the room’s isocenter. Detectors were positioned at 2 cm water equivalent depth, with their stem perpendicular to both the magnetic field lines and the proton beam’s central axis, in the direction of the Lorentz force. Normality and two sample statistical Student’s t tests were performed to assess the influence of the magnetic field on the detectors’ responses.

**Results:**

For all detectors, a small but significant magnetic field‐dependent change of their response was found. Observed differences compared to the no magnetic field case ranged from +0.5% to −0.7%. The magnetic field dependence was found to be nonlinear and highest between 0.25 and 0.5 T for 252.7 MeV proton beams. A different variation of the Farmer chamber response with magnetic field strength was observed for irradiations using lower energy (97.4 MeV) protons. The largest magnetic field effects were observed for plane‐parallel ionization chambers.

**Conclusion:**

Small magnetic field‐dependent changes in the detector response were identified, which should be corrected for dosimetric applications.

## 1 INTRODUCTION

Advanced image‐guidance methods are well established in current radiotherapy practice. The use of MR image guidance, offering superior soft‐tissue contrast without additional imaging dose in comparison to x‐ray imaging, is rapidly increasing in radiation oncology.^1^, ^2^, ^3^ New MR linac systems, allowing online MR image guidance during radiotherapy treatments, recently started clinical operation.^4^, ^5^ For photons, a significant amount of resources was invested into developing suitable dosimetry procedures and ionizing‐radiation detector correction factors for reliable reference and relative dosimetry in the presence of magnetic fields.^6^, ^7^, ^8^, ^9^, ^10^, ^11^, ^12^, ^13^, ^14^, ^15^, ^16^, ^17^, ^18^, ^19^, ^20^ A detailed overview of the current status of reference dosimetry in MR linacs was recently presented by de Pooter.^21^

Proton therapy would be ideally suited to profit from such an advanced imaging modality, especially due to its higher conformity and increased sensitivity to anatomical changes^22^, ^23^, ^24^, ^25^, ^26^, ^27^, ^28^; consequently research toward online MR guidance during proton therapy is gaining momentum.^29^, ^30^, ^31^, ^32^ The high magnetic fields required for imaging impose additional challenges.^33^ In contrast to photon‐based therapy, where only secondary electrons are affected by the magnetic field, the primary particles are also affected in proton therapy.^32^, ^34^, ^35^ The dosimetric impact of these effects for proton therapy was already studied in silico, typically employing Monte Carlo simulations. Strategies to compensate for these effects during dose calculation and treatment planning are currently being developed.^36^, ^37^, ^38^

A prerequisite for the development of a combined MR proton therapy machine is the ability to perform accurate reference and relative dosimetry. However, so far only limited dosimetric measurements have been performed with protons in magnetic fields, mostly employing passive film detectors.^29^, ^31^, ^39^ This is further hampered by the limited availability of proton beam lines equipped with research magnets, as well as the lack of dedicated commercial dosimetry equipment that has been validated for this purpose. Due to the constrained environment of research magnets, current available dosimetry systems for MR linacs are only of limited use.^12^

So far, the suitability of existing detectors for proton dosimetry in magnetic fields was not yet studied. Due to the different radiation properties and secondary particle spectra, it is not straightforward to transfer dosimetric corrections from MR‐guided photon therapy.

In this manuscript, ionizing‐radiation detectors were characterized in reference conditions in water using an in‐house developed phantom. A variety of detector types, typically used to cover a broad range of applications such as reference dosimetry, laterally integrated depth dose, and beam profile measurements, as well as patient specific quality assurance was selected. The response of two plane‐parallel ionization chambers (PPICs), two thimble‐type ionization chambers (ICs), and a diamond detector was evaluated in magnetic fields ranging from 0 to 1 T.

## 2 MATERIALS AND METHODS

### 2.A Measurement setup

A resistive H‐shaped dipole magnet is available in the research irradiation room with a horizontal beam line at MedAustron. The magnet (Danfysik, Taastrup, Denmark) features an air gap of 13.5 cm between the poles with a diameter of 25 cm. The magnetic field strength is adjustable up to 1 T. Magnetic field homogeneity was evaluated by the manufacturer to be within 0.93% of the design value in a sphere of 75 mm diameter around the magnet center. The magnetic field strength falls to ambient levels at a distance of 50 cm of the magnet isocenter. The magnet is mounted on a support system, featuring racks for the power supply system and heavy‐duty wheels to allow easy repositioning. The magnet was positioned such, that the isocenter of the beam coincides with the magnet isocenter.

The research room is equipped with a medical nozzle, fully commissioned according to clinical specifications, allowing quasi‐discrete spot scanning over the clinical nominal energy range of 62.4 to 252.7 MeV for protons and maximum field sizes of 20 × 20 cm^2^. However, to avoid irradiating the magnet, maximum field sizes were limited to 10 cm in height in this study.

Magnetic fields are expected to influence the intrinsic response of some characteristic ionizing‐radiation detectors used in proton beam therapy. It is well known that the secondary electron spectra of a proton beam depend on its energy, with higher average electron energies being observed for higher proton energies. The impact of an applied magnetic field is likely higher with larger secondary electron ranges. Consequently, the highest proton energy available at our accelerator was employed, leading to electron CSDA ranges in water of 2.3 mm at the corresponding measurement depth of 2 cm.^40^

Although less pronounced than for photon‐based dosimetry studies in magnetic fields, a significant electron return effect was measured and reported for protons in magnetic fields.^31^ Special attention was paid in this work to avoid such effects from occurring due to air gaps in addition to detector‐related effects. Therefore, water was chosen as phantom medium.

An in‐house‐designed motorized water phantom was used to position the detectors, assuring that all measurements were conducted within the highly homogeneous magnetic field region. Previous reproducibility and accuracy studies of the phantom positioning system showed uncertainties always lower than 0.2 mm and a maximum deviation of the scale value and the mechanically measured value of 0.3 mm.^41^

**Fig. 1:**
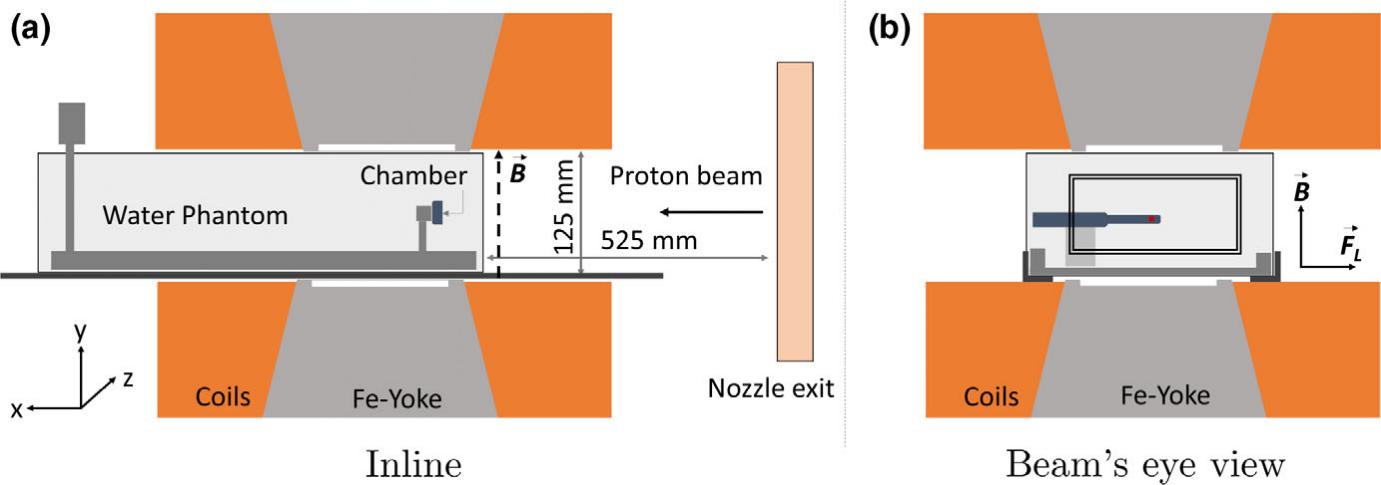
Sketch of the measurement setup, showing the inline (left side) and beam eye view (right side) planes. The orientations of the magnetic field (B) and the corresponding Lorentz force (FL) vectors are also displayed. Proton beams are deflected alongside the positive direction of the z axis. The irradiation field for the Farmer chamber configuration is also highlighted with a rectangle in the right figure.

The PTW TRUFIX detector attachment system (PTW, Freiburg, Germany) was used. In‐house‐developed adapters were used to attach the corresponding TRUFIX holders to the water phantom. All detectors were positioned with their effective point of measurement (EPOM) at a water equivalent depth of $z_{\mathrm{ref}}=2cm$. Detector reference points were offset according to IAEA TRS‐398 recommendations for heavy ion beams,^42^ see Table I. A reference depth of 2 cm is the most commonly used for reference dosimetry in scanned proton beams^43^, ^44^ and also the depth used for clinical commissioning of the beam monitor calibration at MedAustron. Detectors were always oriented with their stem perpendicular to the beam axis and perpendicular to the magnetic field lines, in the direction of the Lorentz force (see Fig. 1). In‐room and secondary lasers were used to align this reference point laterally to the center of the magnet, assuring equivalent irradiation conditions for all the chambers.

**Table I:** Overview of the investigated detectors and employed magnetic field strengths.

Detector	Type	Nominal active volume ( cm^3^)	RPD (cm)
PTW‐30013	Farmer‐type thimble IC	0.600	2.23
PTW‐31016	PinPoint‐type thimble IC	0.016	2.16
PTW‐34001	Roos‐type PPIC	0.350	2.00
PTW‐34073	Bragg peak‐type PPIC	2.500	2.00
PTW‐60016	microdiamond‐type solid state detector	0.004	2.00

The position of the reference point of the detector (RPD) was selected to achieve an effective point of measurement (EPOM) of 2 cm water equivalent depth.

Magnetic fields of 0, 0.25, 0.5, 0.75, and 1 T were employed. Magnetic field strength was verified before the measurements using a portable hall‐probe sensor AS‐NTM connected to a FM 302 Teslameter (Projekt Elektronik Mess‐ und Regelungstechnik GmbH, Berlin, Germany). Deviations between measured and nominal field strengths were always less than 0.2 mT. Stabilization times for the magnetic field strength lower than 2 min after ramping the current were observed to achieve field strengths variations in the order of 0.1 mT. During our measurements, a waiting time in the order of 3 min was given after each change of the magnetic field strength.

#### 2.A.1 Investigated detectors

Detectors covering a representative variety of types and geometries have been selected. The thimble‐type ICs were a PTW‐30013 Farmer type and a PTW‐31016 PinPoint type (both PTW, Freiburg, Germany). The PPICs were a PTW‐34001 Roos type and a PTW‐34073 Bragg peak type (both PTW, Freiburg, Germany). In addition, a PTW‐60019 microDiamond solid‐state detector (PTW, Freiburg, Germany) was investigated. Special care was taken to select detectors with differing dimensions and active volumes, details can be found in Table I. Charge produced in the detectors was read out using a Unidos Webline electrometer (PTW, Freiburg, Germany).

The chambers employed in this investigation were not explicitly certified to be MR compatible by the vendor. To our understanding, potentially problematic parts could be contained in the chamber connection plugs. Consequently, special care was taken to ensure that these parts remained outside of the magnetic field. Before starting the measurements, the unconnected chambers were exposed to the magnetic field and no forces acting on the chambers were detected. Functional tests performed before and after magnetic field exposure were found to yield identical results within the measurement uncertainty, indicating that the magnetic fields had no influence on the chamber performance.

### 2.B Measurements

After alignment, a preirradiation of 15 Gy was performed for every detector. Irradiation fields were adapted for the different detectors, ensuring field sizes extending at least 3 cm outside the detector geometry. Magnetic fields affected the beam path, leading to a lateral position offset of up to 10 mm at the EPOM of the detectors for a 252.7 MeV proton beam. To compensate for this effect, the chamber position was kept constant in all the irradiations, but the irradiation field was laterally enlarged. Single‐energy‐layer fields for 252.7 MeV proton of 10 × 8 cm^2^ and 10 × 10 cm^2^ were created employing a 2 mm spot spacing in both orthogonal directions with a constant spot weighting. The spot spacing was selected to be lower than 25% of the resulting single beam spot sizes at the chamber measuring position, ensuring that the dose delivered to the chambers was highly homogeneous without significant lateral dose gradient in the area occupied by the detectors. A physical dose of 0.21 Gy was delivered at the detector position. To assess the influence of the proton beam energy on the response of the detectors, complementary measurements were performed for the Farmer chamber using a similar single‐energy‐layer irradiation field, but with a lower nominal energy of 97.4 MeV.

All detectors were tested in homogeneous magnetic fields with doubling field strengths of 0.25, 0.5, and 1 T. In addition, for the Roos‐type IC the response was investigated in a magnetic field of 0.75 T. For each detector, irradiations at the different magnetic field strengths were performed in the same setup, with no repositioning. Measurements at each magnetic field strength were always alternated with reference measurements without magnetic field. All investigated field strengths were repeatedly applied, typically measuring five times at each iteration. In total 25–60 measurements were acquired per detector and field strength (the total acquired number of measurements per setting can be found in Table II). Every five irradiations, a leakage charge (~2 pC) correction (electrometer zeroing) was performed. The responses of the ICs were always corrected for temperature and pressure.

**Table II:** Summary of measured data for the five analyzed detectors using 252.7 MeV proton beams.

Chamber	B(T)	#Meas	$k_{B,M,Q}$	SD	Normality test *P*‐value	Student’s *t* test *P*‐value
Roos	0	55	1.000	0.001	0.732	–
	0.25	35	1.005	0.001	0.729	≤0.001*
	0.50	35	1.007	0.001	0.850	≤0.001*
	0.75	35	1.003	0.001	0.348	≤0.001*
	1.00	25	0.999	0.002	0.277	0.002*
Bragg peak	0	46	1.000	0.001	0.711	–
	0.25	25	1.007	0.002	0.371	≤0.001*
	0.50	25	1.008	0.002	0.633	≤0.001*
	1.00	25	1.001	0.001	0.580	0.287
Farmer	0	60	1.000	0.002	0.978	–
	0.25	40	1.003	0.001	0.710	≤0.001*
	0.50	40	1.001	0.002	0.403	0.038*
	1.00	40	0.998	0.002	0.964	≤0.001*
PinPoint	0	55	1.000	0.003	0.395	–
	0.25	35	1.007	0.003	0.671	≤0.001*
	0.50	35	1.007	0.003	0.718	≤0.001*
	1.00	40	1.005	0.003	0.552	≤0.001*
μ Diamond	0	60	1.000	0.008	0.778	–
	0.25	35	1.003	0.008	0.286	0.058
	0.50	35	0.997	0.005	0.968	0.020*
	1.00	40	0.997	0.005	0.654	0.026*

For the Bragg peak chamber, a linear drift correction was applied. Baseline‐corrected data are presented. The Student’s *t* test column displays the statistical significance of a difference to the 0 T field case. Values below the clinical significance threshold of 5% are highlighted using *

### 2.C Data evaluation and statistical testing

According to TRS‐398,^42^ the absorbed dose to water, $D_{w,Q}$, using an IC is obtained as:

(1) $$D_{w,Q}=M_{Q}N_{D,w,Q_{0}}k_{Q,Q_{0}}$$

where Q is the clinical reference beam quality, $Q_{0}$ is the calibration beam quality, $N_{D,w,Q_{0}}$ is the absorbed dose to water calibration coefficient, and $k_{Q,Q_{0}}$ is the beam quality correction factor. In our experiment the raw detector reading $M_{\mathrm{raw}}$ was corrected for temperature and pressure according to TRS‐398. Note that TRS‐398 recommends reference dosimetry to be performed in the SOBP. For scanned beams, however, the beam monitor has to be calibrated for each energy layer in terms of absorbed dose at shallow depth and it has been demonstrated that the same formalism and data can be used (for a review of the literature on this subject, see Palmans and Vatnitsky, 2016.^43^ This is also the approach used for reference dosimetry at MedAustron.^45^

The factor $k_{B,M,Q}$ was applied to Eq. (1), assuming no change in the local dose to water, to account for the change in chamber response due to a magnetic field, defined as:

(2) $$k_{B,M,Q}=\frac{M_{Q}}{M_{Q}^{B}}$$

where $M_{Q}$ and $M_{Q}^{B}$ corresponds to the corrected detector readings with the magnetic field off and on, respectively. The same formalism, without temperature correction, was applied to the diamond detector. Data evaluation was performed on the corrected charges $M_{Q}$ and $M_{Q}^{B}$ directly, without applying further conversions. To enable an easy comparison among the different detectors, their corrected readings, $M_{Q}^{B}$, were normalized to the nonfield corrected reading, $M_{Q}$.

Statistical analysis was performed with OriginPro 2016 (OriginLab Coorporation, Northampton, MA, USA.). Tests were performed for all detectors, accounting for differences of the mean detector response for different magnetic field strengths (0, 0.25, 0.5, and 1.0 T). A two‐sided Student’s t test was used. A significance level of *P* < 0.05 was chosen for all the statistical analysis of our data. The Shapiro–Wilk test was selected to evaluate the data set normality, while the Levene’s test for homogeneity of variances.

## 3 RESULTS

**Fig. 2:**
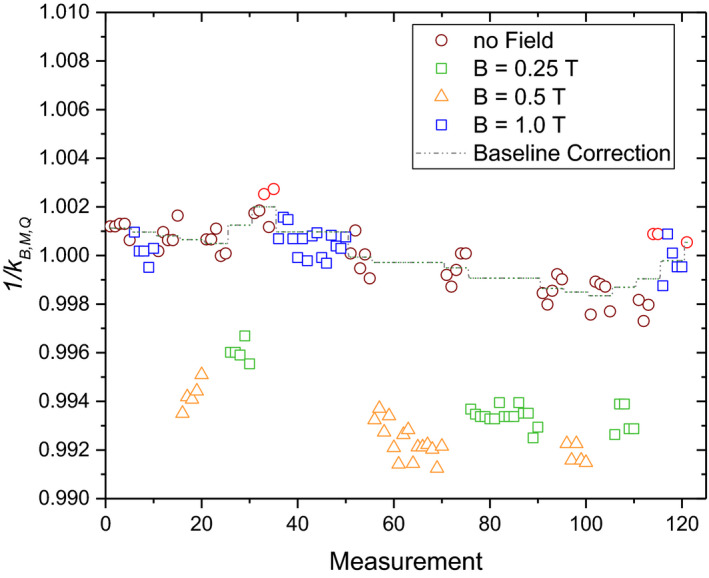
Relative chamber readings of the Bragg peak chamber in water for a 252 MeV proton field of 10 × 10 cm^2^ measured using 0, 0.25, 0.5, and 1 T magnetic field strength. The dashed line indicates the baseline used for drift correction.

All the collected data were normally distributed, except for the Bragg peak chamber, where the detector readings for 0 and 0.25 T showed significant deviations from the normal distribution. Analysis of the collected data showed a drift of the chamber response over time for the Bragg peak chamber (see Fig. 2) and to a lesser extent for the Roos chamber. For the rest of the chambers, no temporal drift was observed. Only for the Bragg peak chamber, a baseline correction was introduced, and the measurements corrected accordingly. The average of five measurements performed at 0 T immediately before and after the corresponding magnetic field measurement was used as corrected baseline.

**Fig. 3:**
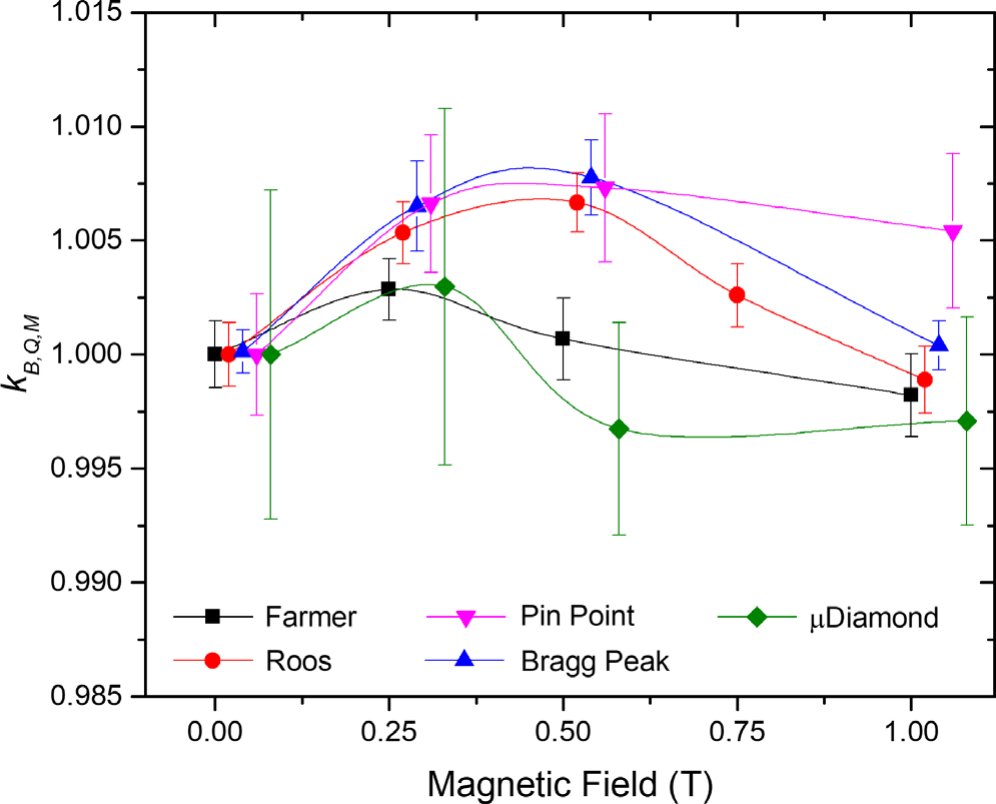
Overall relative chamber response as a function of magnetic field strength for all the detectors used in this study. To increase visibility, data points were shifted along the x axis. All data points were measured at the same magnetic field strengths of 0, 0.25, 0.5, 0.75, or 1 T.

The corrected readings were found to vary based on the detector type and the applied magnetic field strength, with PPICs showing the most prominent dependencies (see Fig. 3). Overall, a change in $k_{B,M,Q}$ values up to 0.7% was found for medium strength magnetic fields, with less differences for higher magnetic field strengths. Statistically significant changes were found for all the analyzed detector responses, but not for all magnetic field strengths.

**Fig. 4:**
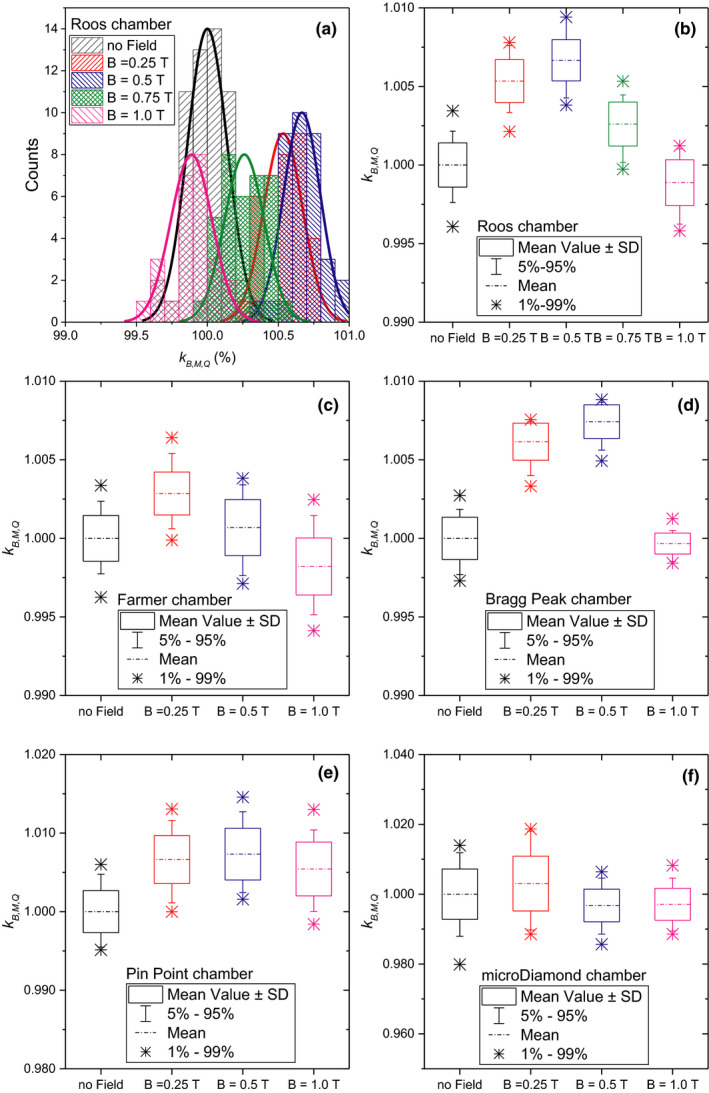
Detailed response values per chamber. (a) distribution of the measurement for the Roos electron chamber; box plots for the Roos electron chamber (b), Farmer chamber (c), Bragg peak chamber (d), PinPoint chamber (e), and the microdiamond chamber (f), respectively.

All detector measurements followed a normal distribution. As an example, the distribution for the Roos chamber is depicted in Fig. 4(a). For PPICs, $k_{B,M,Q}$ increased up to 0.7% for magnetic field strengths until 0.5 T, with a subsequent decreasing for higher magnetic fields. The Roos chamber even exhibited a −0.1% under response at 1 T. The behavior for thimble‐type ICs was similar. The Farmer chamber, when irradiated with 252.7 MeV energy protons, was shown to be less affected by the magnetic fields with an overresponse of 0.3% at 0.25 T and an under response of −0.2% at 1 T. The PinPoint chamber showed overall the highest changes in response of 0.73% for a magnetic field strength of 1 T. Although variations on the detector response were statistically significant for the microDiamond, the measurement variance was comparatively higher, and the differences observed minor. $k_{B,M,Q}$ values, as well as the results for all the statistical tests conducted are summarized in Fig. 4 and Table II, for all the chambers irradiated with 252.7 MeV protons.

**Fig. 5:**
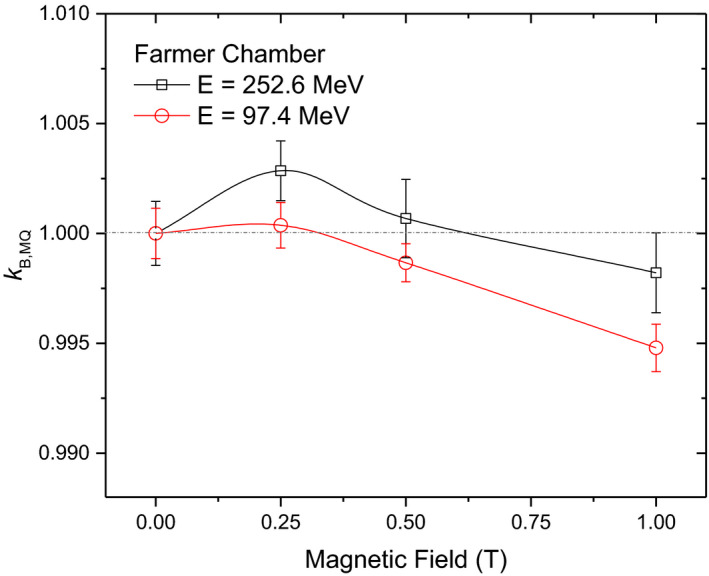
Influence of the magnetic field strength on the Farmer chamber response for irradiations at two different proton beam energies.

Finally, the influence of the initial proton beam energy on the response of the Farmer chamber is depicted in Fig. 5. Results showed a similar trend for the chamber response for both energies, but lower $k_{B,M,Q}$ values were obtained. While at magnetic field strengths up to 0.5 T $k_{B,M,Q}$ is closer to unity for the 97.4 MeV compared to the 252.7 MeV beam, for the lower proton energy $k_{B,M,Q}$ deviates more from unity at higher magnetic field strengths.

## 4 DISCUSSION

For the PPICs small drifts of the measurements were observed over the duration of the experiment. Most likely this drift was due to an interference between the buildup of radiation‐induced charges and repeated zeroing of the electrometer. This was supported by the fact that the drift was reduced after longer irradiation pauses and an additional electrometer zeroing. Only for the Bragg peak chamber, this temporal drift significantly affected the normality of the distribution of the measurements. Consequently, a custom baseline correction was introduced during data analysis. The observed fluctuations for the Bragg peak chamber’s temporal drift prevented the use of a single linear fit for a baseline correction. Recently it was reported that the homogeneity of Bragg peak chambers is not uniform^46^, ^47^ and thus effects seen in this study could be influenced by its heterogeneous response. However, as the observed magnetic field dependency was small and the Bragg peak chamber air gap variance was reported to vary by 5%, such an effect would probably not be noticeable.

A small, but quantifiable, effect was found for all tested ICs. The magnetic field influence on the reading was field‐strength dependent, resulting in the largest correction factors between 0.25 and 0.5 T for irradiations with 252.7 MeV proton beams. The observed effect was less prominent for thimble‐type ICs. For lower energy protons (97.4 MeV), the highest deviation was observed at B = 1T with a difference more than twice compared to the higher proton energy. The variation of the chamber response depended on the magnetic field strength and also on the incident proton beam energy, as expected. The Lorentz force affects the motion of primary and secondary particles, resulting in spiral trajectories with different radius of curvature in the chamber volume. For the same magnetic field strength, lower energy particles will have a smaller radius of curvature. Further studies are planned using different proton beam energies and magnetic field intensities.

The factor $k_{B,M,Q}$ was determined assuming the local dose to water does not change due to influence of the magnetic field on the dose distribution. MC simulations using a validated beam model^48^, ^49^ were employed to support the experiments and compare dose distributions at the chamber measuring position ($z_{\mathrm{ref}}=2cm$). Simulated 3D dose distributions for both 252.7 and 97.4 MeV proton irradiations (averaged in the chamber active volume) showed that the impact of the magnetic field on the dose to water at the measurement point was negligible.

For detectors with the smallest active volume, such as the microdiamond detector or the PinPoint chamber, demonstrating a significant effect of magnetic fields on the chamber responses turned out to be more challenging. Higher variability on the data, attributed to the lower collected charges by the detectors, resulted in standard deviations comparable to the change in the detector responses, see Fig. 3. For easier comparison all detectors were irradiated through this work with the same dose level. However, higher irradiation doses might be necessary for the smaller chambers in order to reduce the measurement variance.

Photon‐based dosimetric studies in magnetic fields^9^, ^13^, ^15^, ^19^, ^20^, ^50^ showed that response curves of the detectors vary differently with the magnetic field strength depending on the relative orientation between the incident beam, the chamber axis, and the magnetic field vector. The impact of magnetic fields on the relative response of ICs exposed to photon beams was recently summarized,^21^ revealing deviations up to 9% as compared to the response in the absence of a magnetic field. In our study, the impact of magnetic fields on chamber readings was considerably lower as compared to photons. A direct back‐to‐back comparison of our results with previously reported data for the MRgRT systems is not trivial. One of the main obstacles is that interaction processes of proton beams passing through media generate different secondary electron spectra, compared to the ones observed for photon beams. Monte Carlo simulations performed within the scope of our research group indicated secondary electron energies up to the initial photon energy (6 MV) for beams used typically in MRgRT systems, resulting in ranges in water in the order of 15–35 mm. Maximum gyroradii of the secondary electrons of 62 and 14 mm were obtained for the two commercial MRgRT systems using magnetic fields of 0.35 and 1.5 T, respectively. In contrast, the secondary electron spectra generated by clinical proton beams comprise energies lower than 0.18, 0.32, and 0.6 MeV for energies of 97.4, 148.2, and 252.7 MeV, respectively. The typical ranges of these secondary electrons in water will be always lower than 2.6 mm and the maximum gyroradii will be lower than 9.5 and 2.2 mm for magnetic field strengths of 0.35 and 1.5 T, respectively. Hence, the transport of secondary electrons in magnetic fields, toward and within the volume of detectors, will be completely different if the electrons were produced by clinical photon vs proton beams. In addition, magnetic fields do not only affect the secondary electrons but also the primary proton beam. Therefore, perturbations of the chamber response can result from an interplay between the electrons’ and the protons’ helical movement within the detector volume. As previously mentioned, dedicated MC simulations are planned to determine $k_{B,M,Q}$ correction factors required for future MRgPT dosimetry, as a comparison with photon‐based previous studies is not straightforward.

Chambers were investigated in the most common orientation typically employed in proton dosimetry. Due to space restrictions in our measurement setup, angular dependencies have not yet been investigated. Another limitation of our study was the limitation of the magnetic field strength to 1 T, imposed by our research magnet. Although our experimental setup may not exactly match the magnetic field’s volumetric dimensions and strengths that can be expected for future MRgPT systems, this study aimed to provide initial data toward reference dosimetry for protons in magnetic field environments.

Through this study, all tested detectors appear to be suitable for proton dosimetry in magnetic fields. The observed overall change in chamber response is small, however, it should be considered toward reference dosimetry in MRgPT.

## 5 CONCLUSION

Five commonly used commercial ionizing‐radiation detectors were investigated concerning their suitability for proton dosimetry in magnetic fields. Experimental data show a statistically significant magnetic field‐dependent influence on chamber response. Although the overall effect is small, it was observed for all investigated ICs and should be corrected for accordingly.

## CONFLICT OF INTEREST

The authors have no conflict to disclose.
